# Climate Variables Outstrip Deadwood Amount: Desiccation as the Main Trigger for *Buxbaumia viridis* Occurrence

**DOI:** 10.3390/plants10010061

**Published:** 2020-12-30

**Authors:** Michaela Kropik, Harald G. Zechmeister, Dietmar Moser

**Affiliations:** 1 Institute of Botany, University of Natural Resources and Life Sciences, 1180 Vienna, Austria; michaela.kropik@univie.ac.at; 2 Department of Botany and Biodiversity Research, University of Vienna, 1030 Vienna, Austria; dietmar.moser@univie.ac.at

**Keywords:** moss, Habitats Directive, conservation, management, biogeographic region, climatic gradient

## Abstract

Deadwood is a biodiversity hotspot and habitat for numerous highly endangered species. *Buxbaumia viridis* has been assessed as a flagship species for deadwood-rich forests and is subject to monitoring under the Habitats Directive, yet we lack a solid understanding of the factors controlling its distribution. The study aimed to specify the climate and habitat preferences of *Buxbaumia viridis* and identify the best predictor variables. We collected presence-absence data of the species at 201 sites between 2016 and 2020. Study sites cover three biogeographic regions (Pannonian, Continental, and Alpine). They also represent a deadwood gradient ranging from managed forests to natural forest reserves and virgin forests. Our results suggest that desiccation and deadwood amount are the best predictor variables. The amount of deadwood at the colonized sites ranged from 1 m^3^/ha to 288 m^3^/ha, with a median of 70 m^3^/ha. The maximum desiccation, i.e., consecutive days without rain and at least 20 °C was 9.6 days at colonized sites. The results of logistic regression models suggest that desiccation limits *Buxbaumia viridis* occurrence on deadwood in the drier continental parts of eastern Austria. Derived details on climate and habitat requirements of *Buxbaumia viridis* can specify management and conservation. They clearly show how strongly the species is dependent on climate, which can counteract deadwood measures.

## 1. Introduction

The epixylic forest bryophyte *Buxbaumia viridis* (Moug. ex Lam. & DC.) Brid. ex Moug. & Nestl. is a flagship species for forests rich in deadwood. It is also subject to the monitoring obligation of the Habitats Directive. While its life cycle [1,2], substrate preferences [3,4,5,6], seasonal variations in occurrence [3,6,7,8], and distribution [9] are well documented, we lack a solid understanding of the factors that control its distribution.

In general, bryophytes react sensitively to climatic parameters [10,11,12,13]. They can even indicate past climate changes [14]. Despite their tolerance to desiccation [15,16,17], the availability of water during their life cycle is of fundamental importance: in the dispersal phase, desiccation restricts the viability of the propagation units; during establishment, it affects the carbon balance of the young plant and limits the period during which establishment is possible [17]. Sensitivity to desiccation varies widely between species [12,16,18], as does the recovery rate [17]. Most bryophytes survive short to moderate periods of desiccation, but many species adapted to a humid, shady habitat are the least tolerant and recover only slowly from long, dry periods [10]. Forest bryophytes can, therefore, be damaged by even moderate desiccation [17].

Furthermore, for the occurrence of *Buxbaumia viridis*, a constant high humidity, which prevents dehydration, was assumed to be one of the most important triggers [3], but no concrete figures are available. Precipitation and temperature in spring influenced the abundance of sporophytes in a local study in Sweden [19], and desiccation in summer reduced spore production [6]. Moreover, the amount of deadwood seems to be decisive for occurrence according to a local study in Italy [4]. However, numerous records in managed forests indicate a wider tolerance for the amount of deadwood [20]. To date, there is no study on a larger geographical scale that considers both climate influence and deadwood quantity, and it remains unclear whether climate or the amount of deadwood is the main trigger for the occurrence of the species. A better understanding is crucial for management decisions, as *Buxbaumia viridis* is an Annex II species of the Habitats Directive and an important flagship species for deadwood-rich forest ecosystems, which are a hotspot of diversity for a wide range of taxonomic groups [21,22].

In consequence, this study aims to specify the *Buxbaumia viridis* preferences in terms of climate and habitat, and to define the main predictors for the observed distribution. The study is based on a large dataset of field observations across the pronounced climatic gradient of Austria considering three biogeographic regions (Pannonian, Continental, and Alpine). It includes data both on climate and deadwood on a large geographical scale for the first time. In addition to standard climate variables, it takes into account several climate variables that have been adapted to the physiological needs of bryophytes.

## 2. Results

**Figure 1:**
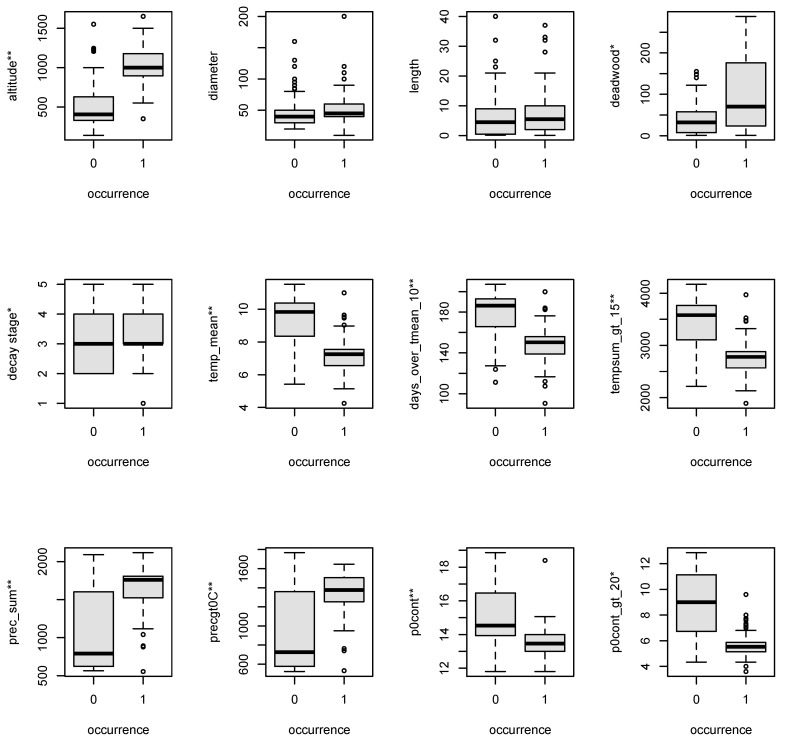
Boxplots for not populated (=0) and populated sites (=1) regarding the climate variables (for abbreviations see Table 1); * and ** give information on the *p*-value’s significance (* = *p* < 0.05, ** = *p* < 0.01).

In total, we found 751 sporophytes of *Buxbaumia viridis* at 278 logs at 115 sites. Altitudes of populated sites ranged between 350–1650 m above sea level (a.s.l.) (median 1000 m). The most common host tree species was *Picea abies* (L.) H.Karst. (184 occurrences) followed by *Fagus sylvatica* L. (42), *Abies alba* Mill. (36), *Larix decidua* Mill. (8), and *Pinus nigra* J.F.Arnold (8). *Buxbaumia viridis* was found at all decay stages [23] between 1 and 5, with the highest frequency in decay stages 3 and 4. We found the species across all forest types, in virgin forests, natural forest reserves, and managed forests. Populated versus not populated sites differed significantly regarding altitude, climate variables, and deadwood amount (Figure 1); only for length and diameter, the difference was not significant.

The maximum number of consecutive days without precipitation and at least 20 °C (p0cont_gt_20) and the amount of deadwood (deadwood) at study site were identified as most important predictors for the occurrence of *Buxbaumia viridis* by a logistic regression model (Table 2). For the sake of simplicity, we refer to these two predictors as desiccation and deadwood in the following.

The effect of desiccation on the occurrence of *Buxbaumia viridis* was significantly negative, while deadwood had a significant positive effect. Regarding the predictive power, desiccation (variable importance 5.949) outstripped deadwood (2.053). Concrete values for desiccation at populated sites varied between 3.6–9.6 days (median 5.5 days), so there were no more than 10 consecutive warm days without rain at these sites. Values for deadwood were between 1–288 m^3^/ha (median 70 m^3^/ha). Multicollinearity was excluded due to variance inflation factors around one and thus far below the generally assumed thresholds. Based on the final model (Table 2), occurrences of *Buxbaumia viridis* were predicted with a sensitivity (i.e., the proportion of correctly predicted occurrences) of 84% and a specificity of 86% (i.e., the proportion of correctly predicted absences) for the test dataset, resulting in a true skill statistic value of 0.72, which can be interpreted as good agreement between training and test data.

## 3. Discussion

### 3.1. Habitat Preferences

Concerning habitat preferences, our data were consistent with previous studies. The altitudes of the populated sites were between 350–1650 m a.s.l. (median 1000 m), similar to other European countries [20]. *Picea abies* in decay stages 3 and 4 was the most common substrate, followed by *Fagus sylvatica* and *Abies alba*. However, we also found the species on other host trees and decay stages, which is consistent with previous studies [1,3,4,6].

In our data, there was no correlation between the volume of logs and the occurrence of *Buxbaumia viridis* as found for other epixylic bryophytes [24]. This is also consistent with our field observations. Under a suitable climate, the species occurred even on small broken off branches. The five most common species associated with *Buxbaumia viridis* were *Herzogiella seligeri* (Brid.) Z.Iwats., *Blepharostoma trichophyllum* (L.) Dumort. var. trichophyllum, *Dicranum scoparium* Hedw., *Nowellia curvifolia* (Dicks.) Mitt., and *Hypnum cupressiforme* Hedw. var. cupressiforme. Barely any liverworts occurred in the close vicinity of *Buxbaumia viridis*. The reason might be that pleurocarpous mosses provide a shadier and wetter shelter for germination and protonemal growth. Tiny liverworts on the other hand often grow in dense mats and might outcompete *Buxbaumia viridis* [3] which has slow protonemal growth [25].

### 3.2. Predictors of the Occurrence of Buxbaumia viridis

Results of the model selection suggest desiccation and deadwood amount as best predictors for *Buxbaumia viridis* occurrence. The higher variable importance of desiccation indicates that climate determines the occurrence stronger than the amount of deadwood. A local study from the Italian Alps [4] derived a deadwood threshold of 48–61 m^3^/ha for *Buxbaumia viridis*. Even though we observed a higher number of occurrences at sites rich in deadwood, numerous occurrences were in managed forests with low amounts of deadwood. Other European studies reported numerous populations in managed forests, too [3,20]. Population dynamics for *Buxbaumia viridis* seem to be strongly determined by climate, as suggested by our models and as found in a local study in Sweden [7].

The dominant role of desiccation in our data is congruent with studies on the physiology of bryophytes, which suggest that changes in moisture-related climate parameters are the main drivers for mosses [10]. In particular, desiccation plays a dominant role both during the dispersal phase, as it limits the viability of the propagation units, and during establishment, as it controls the carbon balance of the young plant and limits the time during which establishment is possible [17]. Canopy species are better adapted to desiccation than understory bryophytes [26]. In general, mosses of shady, humid forests tolerate only limited desiccation [17]. Dilks and Proctor [27] reported a desiccation tolerance for selected forest mosses of four days (*Rhythidiadelphus loreus* (Hedw.) Warnst.) up to nine (*Plagiothecium undulatum* (L. ex Hedw.) Schimp.) or ten (*Hylocomium splendens* (Hedw.) Schimp.) days. In our data, the maximum dry days in one piece at populated sites was 9.6, which is congruent with these experimental results.

Sporophytes generally have a lower temperature optimum than gametophytes and are more susceptible to temperature stress and desiccation [28]. In contrast to other bryophytes, which generally have drought-tolerant spores [16], the spores of *Buxbaumia viridis* are comparatively sensitive and died after only 48 h of dry storage [29]. Our data suggest that longer desiccation events in the drier east of Austria during spring and summer limit the distribution of *Buxbaumia viridis* on deadwood to the Alpine and the north-western part of the Continental Biogeographic Region within Austria.

Occurrences of *Buxbaumia viridis* on the forest ground, like Deme et al. [30] have reported for Hungary, might be dedicated to a drier climate. The forest ground is assumed to buffer desiccation more effectively than a log, which is why bryophytes on the ground might be less subject to climatic variability than on deadwood or stumps [31]. For Austria, records of *Buxbaumia viridis* from the ground are rather the exceptions [32,33].

In the final model, temperature was only included in combination with precipitation, in the form of the desiccation variable. Physiological studies on bryophytes have found a greater tolerance to temperature changes than to changes in moisture-related parameters [10]. However, changes in temperature and precipitation can influence the sensitive competitive balance between vascular plants and bryophytes [34]. Moreover, higher respiration losses have been demonstrated for bryophytes to be due to increasing temperatures [35]. Previous studies also observed a decrease in the species richness of bryophytes as a result of experimental warming in winter and at the same time strongly varying reactions of the individual species [18]. This underlines the need for detailed studies on single species if the impacts of climate change are to be assessed.

### 3.3. Implications for Conservation

Although the current status of *Buxbaumia viridis* has been assessed as stable for Europe [20], the species is vulnerable. Our data suggest its occurrence is closely linked to deadwood under a suitable climate, whereby desiccation and deadwood were the key predictors. The precipitation regime could be altered by climate change [36], as could the tree-communities [37] and thus the quality of available deadwood. Although *Buxbaumia viridis* grew on different host tree species, it showed a preference for spruce, a tree species that will change its area across Europe fundamentally [38].

Furthermore, the quantity of deadwood is decisive. A higher amount of deadwood increases the probability for larger population sizes and in consequence the risk of getting extinct. Removal of deadwood in managed forests, therefore, is a threat for *Buxbaumia viridis*, as it reduces the probability of survivingon a patchy and short-lived substrate. The same is for numerous other rare epixylic bryophytes [20]. Awareness of the importance of deadwood as a diversity hotspot and refuge for rare and threatened species has increased [21,22], but the isolation of deadwood-rich forests at the landscape scale should also be considered as a severe threat for *Buxbaumia viridis* which was assumed to be dispersal-limited [6,8,39] like numerous other rare epixylic bryophytes [40].

Our results underline the importance of studies on both individual species and various taxonomic groups. While numerous studies have focused on the responses of vascular plants to a changing climate, e.g., [41,42], only a few have dealt with bryophytes [43] despite their importance for biogeochemistry [44]. Since bryophytes and vascular plants differ fundamentally in physiology and scale [45], results derived from vascular plants can neither be generalized to bryophytes nor can the reactions of individual bryophyte species be generalized to all bryophytes. Which parameters trigger other epixylic bryophyte species and communities needs further investigation.

## 4. Materials and Methods

### 4.1. Study Area and Field Methods

The sampling design reflects Austria in terms of climate and forest management intensity. The Austrian Federal Forestry Company (ÖBf) and the Austrian Federal Forest Research Centre (BFW) provided an extensive data set on forest habitats. Out of this, we selected 201 study sites. They spanned the maximum possible gradient in terms of mean annual precipitation sum (554.6–2117.5 mm/a) across the country. Sites depict all three biogeographic regions in which Austria has a share (Alpine, Continental, and Pannonian). They further include virgin forests, natural forest reserves (at least 20 years unmanaged), and managed forests, with markedly different deadwood amounts (1–288 m^3^/ha).

We obtained presence-absence data for *Buxbaumia viridis* between 2016 and 2020. We followed a standardized method. At each site, we randomly selectedten logs by a transect method. We chose the first log subjectively in a characteristic area of a site. Every next log was the nearest one in one of the four directions. We documented the absence or presence of *Buxbaumia virids* for each log, including the number of sporophytes per log. We also recorded associated bryophytes species, host tree species, length, and width of logs and decay stage in five categories [23].

*Buxbaumia viridis* is a small, ephemeral species, easy to be overlooked. We standardized detection probability by (1) conducting fieldwork in the optimal period for detecting sporophytes known from previous studies [46,47,48], (2) limiting all the fieldwork to the same persons, well trained over several years on the search for *Buxbaumia viridis,* and (3) a standardized method at all study sites, which provided not only presence but also real absence data, and therefore a comparable and representative subset of logs at each site. We used the historic geographical range [49] to assess if we covered the entire potential habitat with our observations.

### 4.2. Climate Data

We used a highly resolved grid dataset of daily minimum and maximum air temperature [50] and precipitation sum [51] covering Austria at a spatial resolution of 1 km^2^ and a temporal resolution of one day in the period 2008–2018. From this data set, we extracted climate variables (Table 1) for study sites using the programming language Python. Aside from the standard parameters mean annual temperature (temp_mean), temperature sum of days above 15 °C mean temperature (tempsum_gt_15), and the annual precipitation sum (prec sum), we adapted climate variables to the physiology of bryophytes. We calculated the number of days above 10 °C mean temperature (days_over_tmean_10), a temperature at which many forest bryophytes are on a physiological optimum [43,52,53]. We corrected the annual precipitation sum by excluding the snow (precgt0C), as the growth of bryophytes is directly related to water availability [17]. We developed climate variables approaching desiccation, which has been assumed relevant for the occurrence of *Buxbaumia viridis* in previous studies and due to our observations: the maximum number of consecutive days without precipitation (p0cont) and the maximum number of consecutive days without precipitation and at least 20 °C (p0cont_gt_20).

### 4.3. Deadwood Data

The Austrian Federal Forests Company and the Austrian Research Centre for Forests provided data on the amount of lying deadwood at study sites. For study sites where no deadwood data were available, we conducted them following the same method [54].

### 4.4. Statistics

We compared differences between populated and not populated sites regarding the predictors using Wilcoxon rank-sum tests. For explanatory analysis of predictors, we fitted a logistic regression model with a logistic link function to a reduced dataset of 179 entries due to missing deadwood data for 22 sites. Before analysis, we scaled all variables (Table 1) to zero mean and unit variance. To avoid highly correlated variables, we selected the best variable out of each group (Table 1) based on the Akaike information criterion (AIC). We, therefore, added each variable separately to a model and chose the variable delivering the model with the lowest AIC. We tested the resulting subset of variables by the vif-step function of the package usdm [55] and a threshold of vif 5. With the final set of variables, we ran an automated model selection based on the AIC with glmulti [56] and default settings. We calculated variance inflation factors to test the final model for multicollinearity using the vif-function [57] and assessed the predictive power of variables by the varImp-function [58]. We evaluated the final model using the true skill statistics approach (TSS) [59] by repeatedly (10 times) dividing the data set in two parts: one to calibrate the model (80%) and one to evaluate it (20%); we compared predicted occurrences of *Buxbaumia viridis* with observed occurrences using a confusion matrix [58]. We calculated the TSS value as the sum of specificity (i.e., the proportion of correctly predicted absences) and sensitivity (i.e., the proportion of correctly predicted occurrences) minus one. Statistical analyses and figures were conducted in R 3.6.2 [60]. Results are reported against a significance level of 0.05.

## 5. Conclusions

The main findings are as follows. (1) Data suggest the occurrence of *Buxbaumia viridis* primarily determined by climate. Desiccation, i.e., consecutive days without rain and at least 20 °C had a significant negative effect and limited the distribution of the species. The maximum duration of desiccation at populated sites was 9.6 days. (2) Data suggest that the amount and quality of deadwood were of minor relevance compared to the climate. Deadwood amount at populated sites ranged from 1 m^3^/ha to 288 m^3^/ha. (3) Climate variables that we had adapted to the physiological needs of bryophytes performed better than standard climate parameters for *Buxbaumia viridis* and provided consistent results like physiological experiments on forest bryophytes.

## Figures and Tables

**Table 1:** List of climatic variables used in the analysis.

Temperature	Temp_Mean	Mean Annual Temperature
	tempsum_gt_15	temperature sum of days with more than 15 °C mean temperature
	days_over_tmean_10	number of days above 10 °C mean temperature
**Precipitation**	prec_sum	annual precipitation sum
	precgt0C	annual precipitation sum of days with a mean temperature above 0 °C
**Desiccation**	p0cont	maximum number of consecutive days without precipitation
	p0cont_gt_20	maximum number of consecutive days without precipitation and at least 20 °C

**Table 2:** Final model to explain the occurrence of *Buxbaumia viridis* across Austrian; AIC = Akaike information criterion; for abbreviations of climate variables see Table 1.

	Estimate	Std.Error	*z*-Value	*p*-Value
AIC = 173.27; R^2^ = 0.312				
p0cont_gt_20	−0.934	0.157	−5.949	0.000
deadwood	0.009	0.004	2.053	0.040

## Data Availability

The Data are not publicly available as they are an essential part of further publications.
